# DeepCSO: A Deep-Learning Network Approach to Predicting Cysteine S-Sulphenylation Sites

**DOI:** 10.3389/fcell.2020.594587

**Published:** 2020-12-01

**Authors:** Xiaru Lyu, Shuhao Li, Chunyang Jiang, Ningning He, Zhen Chen, Yang Zou, Lei Li

**Affiliations:** ^1^School of Basic Medicine, Qingdao University, Qingdao, China; ^2^College of Life Sciences, Qingdao University, Qingdao, China; ^3^Collaborative Innovation Center of Henan Grain Crops, Henan Agricultural University, Zhengzhou, China; ^4^Key Laboratory of Rice Biology in Henan Province, Henan Agricultural University, Zhengzhou, China; ^5^School of Data Science and Software Engineering, Qingdao University, Qingdao, China

**Keywords:** machine learning, modification site prediction, deep learning, Cysteine S-sulphenylation, post-translational modification

## Abstract

Cysteine S-sulphenylation (CSO), as a novel post-translational modification (PTM), has emerged as a potential mechanism to regulate protein functions and affect signal networks. Because of its functional significance, several prediction approaches have been developed. Nevertheless, they are based on a limited dataset from *Homo sapiens* and there is a lack of prediction tools for the CSO sites of other species. Recently, this modification has been investigated at the proteomics scale for a few species and the number of identified CSO sites has significantly increased. Thus, it is essential to explore the characteristics of this modification across different species and construct prediction models with better performances based on the enlarged dataset. In this study, we constructed several classifiers and found that the long short-term memory model with the word-embedding encoding approach, dubbed LSTM_*WE*_, performs favorably to the traditional machine-learning models and other deep-learning models across different species, in terms of cross-validation and independent test. The area under the receiver operating characteristic (ROC) curve for LSTM_*WE*_ ranged from 0.82 to 0.85 for different organisms, which was superior to the reported CSO predictors. Moreover, we developed the general model based on the integrated data from different species and it showed great universality and effectiveness. We provided the on-line prediction service called DeepCSO that included both species-specific and general models, which is accessible through http://www.bioinfogo.org/DeepCSO.

## Introduction

Protein Cysteine S-sulphenylation (CSO) is the reversible oxidation of protein cysteinyl thiols to suphenic acids. S-sulphenylation functions as an intermediate on the path toward other redox modifications, such as disulfide formation, S-glutathionylation, and overoxidation to sulfinic and sulfonic acids (Paulsen and Carroll, 2013; Huang J.J et al., 2018). This modification has been reported to influence protein functions, regulate signal transduction and affect cell cycle (Van Breusegem and Dat, 2006; Men and Wang, 2007; Paulsen and Carroll, 2013; Hourihan et al., 2016; Choudhury et al., 2017; Mhamdi and Van Breusegem, 2018). So far, thousands of CSO sites have been identified from different species including the mammal *Homo sapiens* and the plant organism *Arabidopsis thaliana* using the chemoproteomics approach (Yang et al., 2014; Li et al., 2016; Gupta et al., 2017; Akter et al., 2018; Huang et al., 2019; summarized in Supplementary Table 1). Nevertheless, the CSO site detection remains a major methodological issue due to low abundance and dynamic level of CSO-containing proteins *in vivo*. In contrast to the time-consuming and expensive experimental approaches, computational methods for predicting CSO sites have attracted considerable attention because of their convenience and efficiency.

Several computational methods have been developed for the prediction of CSO sites, mainly based on a single human dataset containing 1105 identified CSO sites (Yang et al., 2014). They include MDD-SOH (Bui et al., 2016a), iSulf-Cys (Xu et al., 2016), SOHSite (Bui et al., 2016b), PRESS (Sakka et al., 2016), Sulf_FSVM (Ju and Wang, 2018), S-SulfPred (Jia and Zuo, 2017), Fu-SulfPred (Wang et al., 2019), SulCysSite (Hasan et al., 2017), SOHPRED (Wang et al., 2016), and PredCSO (Deng et al., 2018). Out of them, two are based on protein three-dimensional structures, in which PRESS relies on four different protein structural properties (Sakka et al., 2016) whereas PredCSO is an ensemble model that combines bootstrap resampling, gradient tree boosting and majority voting with the 21 features refined out using a two-step feature selection procedure (Deng et al., 2018). The advantage of both classifiers is the inclusion of accurate structural features but their drawback is the limitation of the available structures. The rest classifiers are based on protein sequences. They can be classified into two clusters in terms of model complexity. The first cluster contains four relatively simple models. ISulf-Cys is an SVM (Support Vector Machine)-based classifier with the integration of three features including binary, PSAAP, and AAindex (Xu et al., 2016). SOHSite is an SVM-based classifier with the combined features of position-specific scoring matrix (PSSM) and AAindex (Bui et al., 2016b). SulCysSite is an RF (Random Forest)-based classifier with the integration of multiple features (Hasan et al., 2017) and Sulf_FSVM is an fuzzy SVM classifier using mRMR feature selection from three kinds of features (Ju and Wang, 2018). The second cluster includes four relatively complex models. MDD-SOH contains two-layered SVMs trained with MDDLogo-identified substrate motifs (Bui et al., 2016a). S-SulfPred is an SVM-based classifier with the balanced training dataset established using one-sided selection undersampling for negative samples and synthetic minority oversampling for positive samples (Jia and Zuo, 2017). Fu-SulfPred contains two layers of forest-based structure with the reconstruction of training datasets for data balance (Wang et al., 2019). SOHPRED was built by integrating four complementary predictors (i.e., a naive Bayesian predictor, an RF predictor, and two SVM predictors), each of which was associated with different training features (Wang et al., 2016). In summary, the characteristics of these sequence-based models are the combination of distinct types of features, or/and the balancing of training data, or/and the integration of different classifiers. Although the developed classifiers have made contribution to the prediction of CSO sites, most of them are currently inaccessible. Moreover, there is a lack of prediction tools for the CSO sites of multiple species. With the growing number of CSO sites verified, it is essential to develop species-specific prediction models with high accuracy or even a general model.

Compared to traditional machine-learning (ML) algorithms (e.g., SVM and RF) used in the prediction approaches described above, the deep-learning (DL) architecture is a promising ML algorithm. In the DL algorithm, a suitable representation of the input data can be transformed into highly abstract features through propagating the whole model. Superposition of hidden layers in neural networks can increase the ability of feature extraction, resulting in a more accurate interpretation of latent data patterns. Indeed, several frequently utilized DL models have been recently applied in the field of Bioinformatics, especially the prediction of post-translational modification (PTM) sites. For instance, deep neural networks were utilized for the prediction of protein nitration and nitrosylation sites (Xie et al., 2018), recurrent neural networks (RNNs) were employed for the prediction of lysine Malonylation sites (Chen et al., 2018b) and convolutional neural networks (CNNs) were used for the prediction of phosphorylation sites and crotonylation sites (Wang et al., 2017; Zhao et al., 2020). Deep learning algorithms have demonstrated their advantages in the application of large data sets, compared to the traditional ML methodology (Chen et al., 2018b). Because of this, the introduction of DL algorithms into the prediction of CSO sites would be a promising move to provide reliable candidates for further experimental consideration.

In this study, we constructed a number *in silico* approaches for the prediction of the CSO sites for *H. Sapiens* and *A. thaliana*. These approaches included the RF and SVM algorithms, one-dimensional CNN (1D-CNN), two-dimensional CNN (2D-CNN) and long short-term memory (LSTM) that is an RNN type. The LSTM model with the word-embedding encoding approach, called LSTM_*WE*_, compared favorably to the rest approaches with AUC as 0.82 and 0.85 in human and Arabidopsis in terms of cross-validation. Moreover, LSTM_*WE*_ trained using the data from one organism achieved outstanding performance in predicting CSO sites of other organisms (e.g., AUC = 0.80 for the prediction of Arabidopsis CSO sites using the human model), suggesting that CSO is highly conserved. Therefore, we constructed a general CSO prediction model. These models will facilitate the discovery of new CSO sites and thus will contribute to the understanding of roles and functions of CSO in diverse cellular processes.

## Materials and Methods

### Data Collection and Preprocessing

**FIGURE 1:**
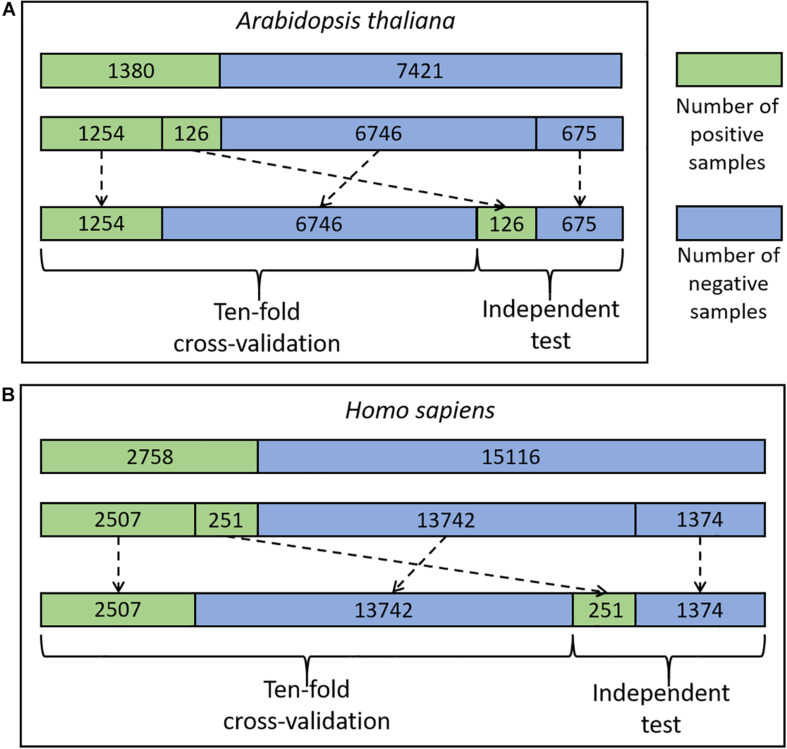
The flowchart of the dataset process for *A. thaliana* **(A)** and *H. sapiens* **(B)**.

The experimentally identified CSO sites were derived from two different organisms including *H. Sapiens* and *A. thaliana* (Yang et al., 2014; Li et al., 2016; Gupta et al., 2017; Akter et al., 2018; Huang et al., 2019). The data of the species were pre-processed and the related procedure was exemplified using the *A. thaliana* data, as listed below (Figure 1A).

We mapped 1537 Arabidopsis CSO sites (Huang et al., 2019) to the UniprotKB database (UniProt Comstortium, 2011) and 1535 sites from 1130 proteins were retained as positive sites. The rest 8819 Cysteine residues in the same proteins were defined as negative sites. Moreover, we truncated these protein sequences into 35-residue segments with the Cysteine located at the center and the positive/negative sites correspond to positive/negative segments, respectively. It should be noted that if the central Cysteine was located around the N-terminus or C-terminus of a protein sequence, the gap symbol “-” was added to the corresponding positions to ensure that the segment had the same length. The segment length was optimized as a hyper-parameter in the Bayesian optimization method (see details in Section of “Optimization Methods for Hyper-Parameters”) and finally determined as 33. Furthermore, to reduce the potential influence of the segments with high similarity on the performance of the models to be constructed, we set the identity of any two sequences with less than 40%, referring to previous studies (Bui et al., 2016a; Wang et al., 2016; Xu et al., 2016). When the identity was >40% between two positive segments or two negative segments, one was randomly removed. When the identity was >40% between a positive segment and a negative segment, the positive was retained and the negative was discarded. As a result, 1380 positives and 7421 negatives were retained. Finally, we randomly separated the positive and negative segments into 11 groups of which 10 were used for 10-fold cross-validation (1254 positives and 6746 negatives) and the rest for an independent test (126 positives and 675 negatives) (Figure 1A). Similarly, the cross-validation dataset for *H. sapiens* contained 16,249 samples (2507 positives and 13,742 negatives) and the independent test set comprised 1625 samples (251 positives and 1374 negatives) (Figure 1B). These datasets are available at http://www.bioinfogo.org/DeepCSO/download.php.

### Feature Encoding Schemes

#### Numerical Representation for Amino Acids (NUM)

The NUM encoding approach maps each type of amino acid residue to an integer (Zhang Y. et al., 2019). Specifically, in the alphabet “AVLIFWMPGSTCYNQHKRDE-”, each letter from “A” to “-” is converted to the integers from 0 to 20 in turn. For example, the sequence “VAMR” is encoded as “1,0,6,17.” This encoding was used as the input of the first layer for both LSTM and 1D-CNN.

#### Enhanced Amino Acid Composition

The enhanced amino acid composition (EAAC) encoding (Chen et al., 2018b,c, 2020; Huang Y. et al., 2018) introduces a fixed-length sliding window based on the encoding of amino acid composition (AAC), which calculates the frequency of each type of amino acid in a protein or peptide sequence (Bhasin and Raghava, 2004). EAAC is calculated by continuously sliding a fixed-length sequence window (using the default value 5) from the N-terminus to the C-terminus of each peptide. The related formula is listed below:

$$f\,\left(t,w\,i\,n\right)=\frac{N\,\left(t,w\,i\,n\right)}{N\,\left(w\,i\,n\right)},t\in \left\{A,C,D,\ldots ,Y\right\},$$

(1) w⁢i⁢n∈{w⁢i⁢n⁢d⁢o⁢w⁢1,w⁢i⁢n⁢d⁢o⁢w⁢2,…,w⁢i⁢n⁢d⁢o⁢w⁢35}

where *N(t, win)* is the number of amino acid *t* in the sliding window *win*, and *N(win)* is the size of the sliding window *win*.

#### Binary Encoding

In the binary encoding (Chen et al., 2018c), each amino acid is represented by a 21-dimensional binary vector that represents 20 amino acids and a complement “-.” The corresponding position is set as 1 and the rest position is set as 0. For example, the amino acid “A” is represented by “100000000000000000000,” “V” is represented by “010000000000000000000,” and the symbol “-” is represented by “000000000000000000001,” according to the alphabet “AVLIFWMPGSTCYNQHKRDE-.”

#### AAindex Encoding

AAindex is a database of various indices representing distinct physicochemical and biochemical properties of amino acids and pairs of amino acids.^1^ In the 544 physicochemical properties, we retained 531 properties after the removal of properties with “NA.” We calculated the performance for each property using the RF classifier based on the 10-fold cross-validation dataset of arabidopsis. We selected the top 36 properties with AUC > 0.7 (Supplementary Table 3).

#### The Composition of k-Spaced Amino Acid Pairs

The composition of k-spaced amino acid pairs (CKSAAP) encoding contains the frequency of the amino acid pair of which both are separated by k-residues (k = 0, 1, 2, 3, 4, 5. We used the default value 5) (Chen et al., 2018c). This scheme represents the short- or long-range interactions amongst the residues along the sequence. The CKSAAP encoding with k = 0 is identical to the di-peptide composition.

#### The Position-Specific Scoring Matrix

The PSSM encoding was derived from the previous publication (Xie et al., 2018). In brief, we calculated the statistical significance of the differences in the frequencies of symbol occurrence between the positive and negative samples using a two-sample *t*-test (Vacic et al., 2006). Accordingly, the PSSM of significant *P*-values were constructed. By integrating the PSSM of *P*-values with the frequency PSSM for positive and negative samples, we generated the final encoding PSSM that represented the conservation tendency of the positive or negative samples.

### Architecture of the Machine-Learning Models

#### The LSTM Model With the Word Embedding Encoding (LSTM_*WE*_)

**FIGURE 2:**
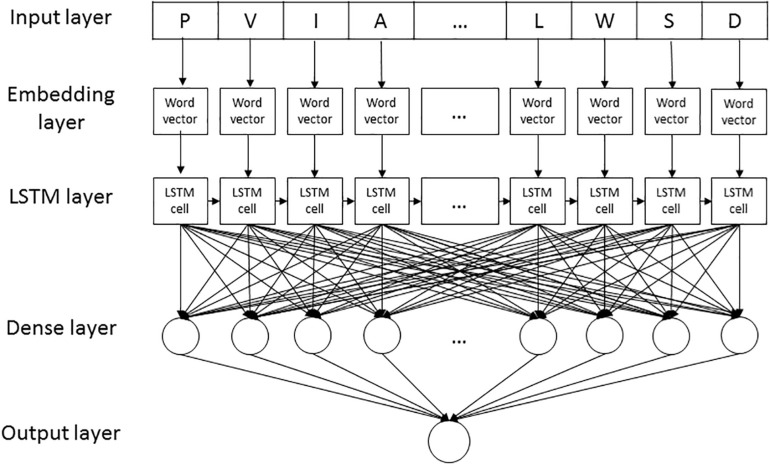
The LSTM_*WE*_ architecture.

LSTM_*WE*_ contained five layers, listed as follows (Figure 2).

1. Input layer. Each peptide segment is converted into an integer vector with the NUM encoding.
2. Word Embedding (WE) layer. Each integer of the vector from the input layer is encoded into a four-dimension word vector for humans and a five-dimensional word vector for arabidopsis, respectively.
3. LSTM layer. Each of the word vectors is input sequentially into the LSTM cell that contained 32 hidden neuron units.
4. Dense layer. It contains a single dense sublayer that has 16 neurons with the ReLU activation function for humans and 32 neurons for arabidopsis, separately.
5. Output layer. This layer has only one neuron activated by sigmoid function, outputting the probability of the CSO modification.

#### The 1D-CNN Model With the Word Embedding Encoding

The 1D-CNN model with the word embedding encoding (1D-CNN_*WE*_) contains five layers (Supplementary Figure 1), where the first two layers and last one layer were as same as LSTM_*WE*_. The third layer was a 1D convolution layer with 22/20 filters for humans/arabidopsis and kernel size as nine. The fourth layer had a single dense sublayer with 16 neurons. The optimal hyper-parameter values were obtained using the Bayesian optimization algorithm.

#### The 2D-CNN Model With the PSSM Feature

We took advantage of the 2D structure of an input image of CNN architecture and conveniently made similar 2D inputs of PSSM matrixes with the sizes of 20 × 20 s. The purpose of using the 2D-CNN model is to catch the hidden figures inside PSSM profiles. Next, PSSM profiles were connected to the 2D CNN design from the input layer through several hidden layers to the output layer. Supplementary Figure 2 demonstrated the procedure of inputting a PSSM profile into the CNN model, then passing through a series of convolutional, non-linearity, pooling and fully connected layers and finally outputting the result. This model contained four hidden layers including one 2D convolutional layer, one pooling layer, one flattening layer, and one fully connected layer. Specifically, the first layer contained a PSSM profile on which we applied 2D convolutional operations with some existing parameters including 5 × 5 kernel size, 15 filters and 1 × 1 stride.

#### The RF Algorithms With Different Features

The RF algorithm integrates multiple decision trees and chooses the classification with the most votes from the trees. Each tree depends on the values of a random vector sampled independently with the same distribution for all trees in the forest. In this study, we constructed the RF models with six different features, including binary encoding, EAAC encoding, AAindex encoding, CKSAAP encoding, PSSM encoding, and WE. The number of decision trees was selected as 580 *via* the grid search method. These classifiers were developed based on the Python module “sklearn.”

#### The SVM Algorithms With Different Features

We applied the Python-based machine learning package “scikit-learn” to implement the SVM algorithm and adopted the “RBF” kernel function to build the SVM models. The above encoding schemes for RF were applied to the SVM model. In particular, we normalized the feature values that do not range between 0 and 1 (such as PSSM) before inputting the SVM model.

### Model Training Strategy

#### Optimization Methods for Hyper-Parameters

**FIGURE 3:**
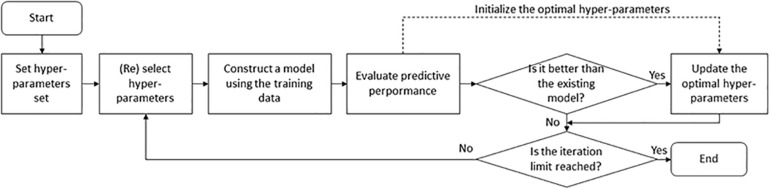
Hyper-parameter optimization procedure for machine-learning classifiers.

The hyper-parameters of an ML classifier affect prediction performance. Although a lot of combinations of hyper-parameters need to be tested, there are no formal rules to find optimal hyper-parameters. Here we applied two search approaches [grid search and Bayesian optimization (BO)] to automatical adjustment and evaluation of hyper-parameters (Figure 3). Grid search is a brute-force method to find the optimal hyper-parameters by training models using each possible combination of hyper-parameters and retaining the hyper-parameters corresponding to the model with the best performance. This method applies to a limited number of hyper-parameters due to the exponential increase in time spent with the number of hyper-parameters. In this study, it was used for the RF-based and SVM-based models. The related grid search spaces (Supplementary Table 3) were searched using the GridSearchCV function of the sklearn library in Python. On the contrary, BO provides a principled technique based on Bayes theorem to direct a search of a global optimization problem, which is effective to tune the hyper-parameters of DL models. The BO strategy was executed using the fmin function of the hyperopt library in Python. The BO related hyper-parameter space contained 10 parameters, including window size, kernel size, and dropout rate (Supplementary Table 3). The optimal hyper-parameter combination results for the DL models were listed in Supplementary Table 4.

#### Strategy of Avoiding Overfitting

1The parameters in the DL models were trained and optimized based on binary cross-entropy loss function using the Adam algorithm. The maximum of the training cycles was set through the optimized number of epochs to ensure that the loss function value converged. In each epoch, the training dataset was separated with the batch size as 512 and iterated. To avoid overfitting, the early-stopping strategy was applied, where the training process was stopped early when the training loss did not go down within 50 consecutive iterations. The model with the smallest training loss was saved as the best model. Moreover, the dropout rate of the neuron units was set, which was obtained through the hyper-parameter optimization. Supplementary Figures 3, 4 showed the training and validation accuracy and loss curves of the LSTM_*WE*_ models for different species.

**FIGURE 4:**
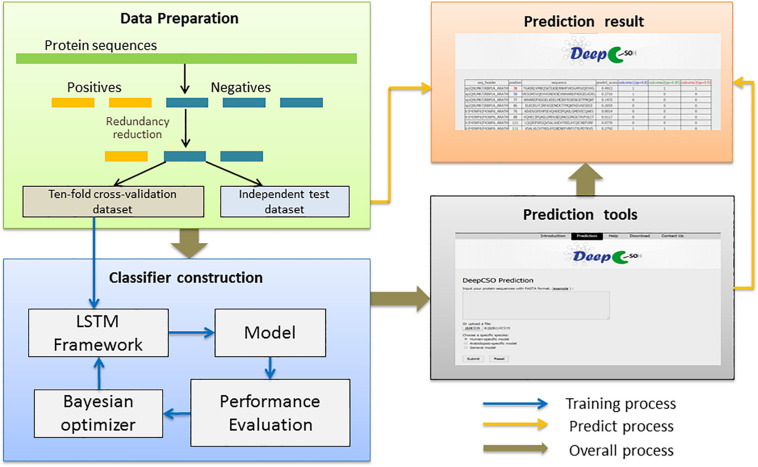
The flowchart of the prediction model construction.

### Performance Assessment of the Predictors

Several measures were used to evaluate the prediction performance, including accuracy (ACC), specificity (SP), sensitivity (SN), Matthew’s correlation coefficient (MCC). They are defined as follows:

$$\text{ACC}=\frac{\text{TP}+\text{TN}}{\text{TP}+\text{FP}+\text{TN}+\text{FN}}$$

$$\text{SP}=\frac{\text{TN}}{\text{TN}+\text{FP}}$$

$$\text{SN}=\frac{\text{TP}}{\text{TP}+\text{FN}}$$

$$\text{MCC}=\frac{\left(\text{TP}\times \text{TN}\right)-\left(\text{FN}\times \text{FP}\right)}{\sqrt{\left(\text{TP}+\text{FN}\right)\times \left(\text{TN}+\text{FP}\right)\times \left(\text{TP}+\text{FP}\right)\times \left(\text{TN}+\text{FN}\right)}}$$

where TP, TN, FP, and FN represent true positives, true negatives, false positives, and false negatives, respectively. Additionally, because the number of positive and negative samples was unbalanced and the above measures were calculated based on the threshold value, a measure that was independent of the threshold value and unaffected by the sample ratio was needed. Therefore, the receiver operating characteristic (ROC) curve and AUC were employed to comprehensively evaluate classification performance. Specifically, due to the low false-positive rate of a predictor is significant in practical application, the area under the ROC curve with <10% false-positive rate (AUC01) was considered.

### Statistical Methods

The paired student’s *t*-test was used to test the significant difference between the mean values of the two paired populations. The adjusted *P*-value with the Benjamini-Hochberg (BH) method was adopted for multiple comparisons.

### The Flowchart of the Prediction Model Construction

The flowchart of the prediction model construction contained three steps (Figure 4). This first step was data collection and preprocessing, in which the sample data were separated into the cross-validation dataset and the independent test dataset for model construction and evaluation. The second step was classifier construction, which involved data decoding, model training, and hyper-parameter adjustment for resulting in a robust predictive model. The third step was the development of the final model as an online prediction tool.

## Results and Discussion

### LSTM_*WE*_ Classifier Performed Favorably to Other Classifiers

Many computational approaches for predicting PTM sites are generally based on traditional ML algorithms (e.g., RF and SVM) combined with various features encoded from peptide sequences. In this study, we constructed both RF-based and SVM-based predictors with different encoding schemes for the CSO site prediction. The encoding schemes include six features [i.e., binary, AAindex, WE, KSAAP, PSSM, and EAAC]. Moreover, deep learning algorithms have recently been applied to the field of PTM site prediction and demonstrated their superior performances (Wang et al., 2017; Chen et al., 2018b). Accordingly, we developed three different DL classifiers, named 1D-CNN_*WE*_, 2D-CNN_*PSSM*_, and LSTM_*WE*_.

We first took the Arabidopsis data to construct and compare different models (Huang et al., 2019). The Arabidopsis cross-validation dataset contained 8000 samples (1254 positives and 6746 negatives) and the independent test set covered 801 samples (126 positives and 675 negatives) (Figure 1). We compared the performances of these algorithms in terms of several measures (e.g., ACC, MCC, AUC, and AUC01) for both the 10-fold cross-validation (Table 1) and the independent test (Supplementary Table 5). In the traditional ML models, RF_*EAAC*_ showed superior performance than other RF-based and SVM-based models. The previous studies of CSO site prediction showed that the models with the combination of different encoding methods compared favorably to their counterparts with a single encoding approach (Bui et al., 2016b; Xu et al., 2016). Accordingly, we constructed such models and the RF model with the combination of EAAC, CKSAAP, and AAindex, dubbed RF_*E+C+A*_, had the best performance. To our surprise, RF_*E+C+A*_ had inferior performance compared to RF_*EAAC*_ (Table 1 and Supplementary Table 5).

**TABLE 1:** Performances of various classifiers for different species in terms of 10-fold cross-validation.

**Classifier^1^**	**ACC^2^**	**Sn^2^**	**Sp^2^**	**MCC2^2^**	**AUC^2^**	**AUC01^2^**
***Arabidopsis thaliana***						
RF_*BINARY*_	0.743 ± 0.006	0.449 ± 0.040	0.798 ± 0.001	0.210 ± 0.032	0.696 ± 0.021	0.014 ± 0.002
RF_*EAAC*_	0.773 ± 0.007	0.628 ± 0.043	0.799 ± 0.001	0.351 ± 0.033	0.803 ± 0.019	0.024 ± 0.004
RF_*WE*_	0.748 ± 0.007	0.474 ± 0.048	0.799 ± 0.001	0.230 ± 0.038	0.728 ± 0.020	0.014 ± 0.002
RF_*AAINDEX*_	0.744 ± 0.008	0.443 ± 0.053	0.800 ± 0.001	0.206 ± 0.043	0.710 ± 0.025	0.014 ± 0.004
RF_*CKSAAP*_	0.749 ± 0.012	0.477 ± 0.078	0.800 ± 0.001	0.234 ± 0.062	0.728 ± 0.032	0.013 ± 0.003
RF_*PSSM*_	0.740 ± 0.006	0.419 ± 0.039	0.800 ± 0.000	0.188 ± 0.032	0.670 ± 0.028	0.015 ± 0.004
RF_*E+C+A*_	0.760 ± 0.006	0.544 ± 0.040	0.800 ± 0.001	0.287 ± 0.031	0.770 ± 0.016	0.020 ± 0.005
SVM_*BINARY*_	0.748 ± 0.009	0.479 ± 0.055	0.798 ± 0.003	0.234 ± 0.043	0.719 ± 0.025	0.017 ± 0.002
SVM_*EAAC*_	0.746 ± 0.009	0.458 ± 0.060	0.799 ± 0.001	0.218 ± 0.048	0.704 ± 0.026	0.015 ± 0.004
SVM_*AAINDEX*_	0.750 ± 0.008	0.486 ± 0.054	0.800 ± 0.000	0.241 ± 0.042	0.724 ± 0.023	0.016 ± 0.004
SVM_*CKSAAP*_	0.739 ± 0.007	0.421 ± 0.047	0.798 ± 0.003	0.187 ± 0.037	0.692 ± 0.030	0.013 ± 0.003
SVM_*PSSM*_	0.726 ± 0.008	0.330 ± 0.054	0.800 ± 0.001	0.113 ± 0.046	0.590 ± 0.025	0.009 ± 0.003
2D-CNN_*PSSM*_	0.766 ± 0.010	0.585 ± 0.064	0.800 ± 0.000	0.319 ± 0.050	0.781 ± 0.030	0.023 ± 0.004
1D-CNN_*WE*_	0.783 ± 0.006	0.696 ± 0.041	0.799 ± 0.001	0.401 ± 0.030	0.838 ± 0.019	0.029 ± 0.005
**LSTM_*WE*_**	**0.786 ± 0.007**	**0.717 ± 0.044**	**0.799 ± 0.001**	**0.417 ± 0.032**	**0.852 ± 0.018**	**0.030 ± 0.006**
***Homo sapiens***						
RF_*BINARY*_	0.749 ± 0.004	0.466 ± 0.027	0.800 ± 0.000	0.225 ± 0.021	0.720 ± 0.013	0.016 ± 0.002
RF_*EAAC*_	0.766 ± 0.006	0.578 ± 0.039	0.800 ± 0.000	0.312 ± 0.030	0.790 ± 0.018	0.020 ± 0.002
RF_*WE*_	0.751 ± 0.004	0.480 ± 0.024	0.800 ± 0.000	0.236 ± 0.019	0.732 ± 0.015	0.018 ± 0.001
RF_*AAINDEX*_	0.750 ± 0.004	0.474 ± 0.025	0.800 ± 0.000	0.231 ± 0.020	0.734 ± 0.017	0.018 ± 0.003
RF_*CKSAAP*_	0.753 ± 0.003	0.493 ± 0.018	0.800 ± 0.000	0.246 ± 0.014	0.729 ± 0.016	0.016 ± 0.002
RF_*PSSM*_	0.748 ± 0.004	0.462 ± 0.026	0.800 ± 0.000	0.222 ± 0.021	0.707 ± 0.016	0.016 ± 0.001
RF_*E+S+A*_	0.761 ± 0.005	0.551 ± 0.033	0.800 ± 0.000	0.291 ± 0.026	0.774 ± 0.012	0.021 ± 0.002
SVM_*BINARY*_	0.750 ± 0.005	0.474 ± 0.030	0.800 ± 0.000	0.231 ± 0.024	0.720 ± 0.013	0.017 ± 0.002
SVM_*EAAC*_	0.742 ± 0.007	0.421 ± 0.049	0.800 ± 0.000	0.188 ± 0.039	0.680 ± 0.021	0.013 ± 0.002
SVM_*AAINDEX*_	0.753 ± 0.006	0.498 ± 0.041	0.800 ± 0.000	0.250 ± 0.032	0.737 ± 0.021	0.017 ± 0.001
SVM_*CKSAAP*_	0.737 ± 0.005	0.388 ± 0.031	0.800 ± 0.000	0.162 ± 0.025	0.664 ± 0.012	0.012 ± 0.002
SVM_*PSSM*_	0.725 ± 0.005	0.316 ± 0.033	0.800 ± 0.000	0.101 ± 0.028	0.578 ± 0.025	0.011 ± 0.002
2D-CNN_*PSSM*_	0.766 ± 0.004	0.581 ± 0.029	0.800 ± 0.000	0.314 ± 0.022	0.777 ± 0.011	0.022 ± 0.003
1D-CNN_*WE*_	0.778 ± 0.006	0.659 ± 0.036	0.800 ± 0.000	0.373 ± 0.027	0.819 ± 0.012	0.024 ± 0.003
**LSTM_*WE*_**	**0.777 ± 0.006**	**0.651 ± 0.038**	**0.800 ± 0.000**	**0.367 ± 0.028**	**0.822 ± 0.011**	**0.024 ± 0.003**

*^1^The RF classifiers with the different features were named as RF_*BINARY*_, RF_*WE*_, etc. The 1D CNN and LSTM classifiers with the word embedding approach were named as 1D-CNN_*WE*_ and LSTM_*WE*_, respectively.^2^ACC, Sn, Sp, MCC, AUC, and AUC01 were described in section “Materials and Methods.” In the 10-fold cross-validation, 10 models were constructed using the 10 different validation datasets. Finally, the average performance and standard deviation of the 10 models were calculated for the cross-validation dataset. The models with the best performances were highlighted in bold.*

All the models constructed above were based on the imbalanced dataset. To evaluate the effect of the imbalanced dataset on potential overfitting of the classifiers, we reconstructed RF_*EAAC*_ based on the balanced positive and negative samples. Specifically, because the number of negative samples was around five times larger than that of the positive samples, we randomly separated the negative samples into five parts and created five subsets of training data with a 1:1 positive-to-negative ratio. Subsequently, five RF_*EAAC*_ models (sub-classifiers) were trained and the average output score from the five sub-classifiers was taken as the final prediction score. Supplementary Figure 5 showed the performances of the two RF_*EAAC*_ models based on the balanced and imbalanced dataset, respectively, in terms of the 10-fold cross-validation and the independent test dataset. Because of the slightly better performance of the RF_*EAAC*_ model constructed using an imbalanced training dataset, we selected the imbalanced dataset for the construction of the models.

**FIGURE 5:**
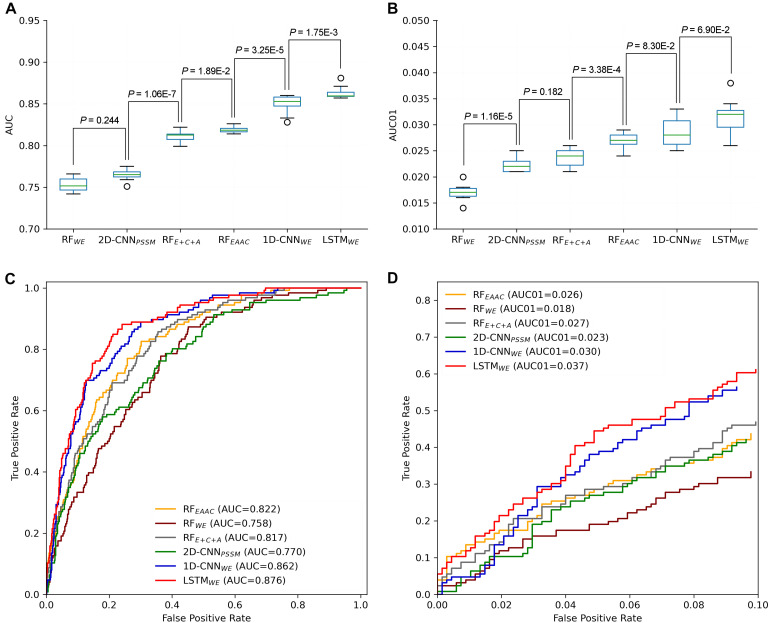
Performance comparison of different CSO predictors on *Arabidopsis thaliana*. The performances of CSO predictors were compared in terms of AUC **(A)** and AUC01 **(B)**, respectively, for 10-fold cross-validation. AUC **(C)** and AUC01 **(D)** curves were generated using the independent test.

In our previous studies, DL models showed superior performance than traditional ML models (Chen et al., 2018b; Zhao et al., 2020). It is still true for the CSO site prediction. LSTM_*WE*_ had the best performance among these constructed models in terms of ACC, Sn, MCC, and AUC values for both 10-fold cross-validation and independent test. For instance, its AUC value is 0.852 for the cross-validation and its values of ACC, Sn, Sp, and MCC were 0.786, 0.717, 0.799, and 0.417, respectively (Table 1 and Figures 5A,C). As prediction performance at a low false-positive rate is highly useful in practice, we estimated these predictors using AUC01, where the specificity was determined to be >90%. LSTM_*WE*_ again showed the largest AUC01 values for both 10-fold cross-validation and the independent test (Figures 5B,D). As the encoding approach has a great impact on the traditional ML models (Chen et al., 2018b; Huang Y. et al., 2018; Zhao et al., 2020) and the WE approach integrated with LSTM had the best performance in this study, we attempted to investigate whether the integration of WE and RF had a good performance. Accordingly, we extracted WE layer vector as feature encoding from LSTM_*WE*_ and trained the RF model, dubbed RF_*WE*_. Interestingly, RF_*WE*_ did not show good performance compared to RF_*EAAC*_, 1D-CNN_*WE*_, or LSTM_*WE*_. It suggests that the WE encoding approach may be improper for the construction of traditional ML algorithms.

We further constructed the models for the human organism. The Humans cross-validation dataset contained 16,249 samples (2507 positives and 13,742 negatives) and the independent test set covered 1625 samples (251 positives and 1374 negatives) (Figure 1B). Similarly, LSTM_*WE*_ had the best performance (Table 1, Supplementary Table 5, and Supplementary Figure 6). For instance, its values of AUC, ACC, Sn, Sp, MCC, and AUC01 for the 10-fold cross-validation were 0.822, 0.777, 0.651, 0.800, 0.367, and 0.024, respectively. We evaluated the robustness of LSTM_*WE*_ by comparing their performances between the cross-validation and independent tests for individual organisms. As their performances were not statistically different for each organism (*P* = 0.18/0.085 for the arabidopsis/humans, respectively), we concluded that the constructed models were robust and neither over-fitting nor under-fitting.

### LSTM_*WE*_ Performed Better Than Reported Classifiers

Six approaches for the prediction of human CSO sites were based on 1105 identified human CSO sites (Yang et al., 2014), including MDD-SOH, SOHSite, SOHPRED, iSulf-Cys, SulCysSite, and Sulf_FSVM. We compare these models and our models (i.e., RF_*EAAC*_, RF_*E+C+A*_, and LSTM_*WE*_) to evaluate their prediction performances. Accordingly, we constructed our models using the same dataset derived from the original study (Yang et al., 2014). SulCysSite, LSTM_*WE*_, and RF_*E+C+A*_ had the best and similar performances (Table 2). The observation that the model with the combined features (i.e., RF_*E+C+A*_) had better accuracy than the counterpart with a single feature (i.e., RF_*EAAC*_) is consistent with the previous studies (Bui et al., 2016b; Xu et al., 2016) but conflicted with our observation above that RF_*EAAC*_ compared favorably to RF_*E+C+A*_. This contradiction derived from the different amounts of the training datasets, where the dataset here was smaller than the datasets described above, indicating that the amount of training data affected the performance of the models. Indeed, based on the small human dataset (1105 positives), RF_*E+C+A*_ had a better performance than RF_*EAAC*_, whereas the performance of RF_*EAAC*_ was better than that of RF_*E+C+A*_ with a large amount of the training set (arabidopsis: 1380 positives; human 2758 positives) (Supplementary Figure 7). In all comparisons, LSTM_*WE*_ showed the best performance (Supplementary Figure). Additionally, as iSulf-Cys (Xu et al., 2016) is the only accessible model to date, we compared it and LSTM_*WE*_ using the human independent dataset of this study. The AUC value (0.839) of LSTM_*WE*_ is significantly larger than that (0.666) of iSulf-Cys (Supplementary Figure 8). In summary, LSTM_*WE*_ performed better than reported classifiers.

**TABLE 2:** The k-fold cross-validation results of existed tools.

Tools*	**Fold**	**Accuracy**	**Sensitivity**	**Specificity**	**AUC**
MDD-SOH	5	0.68	0.7	0.7	
SOHSite	5	0.71	0.72	0.72	
SOHPRED	5		0.727 ± 0.005	0.742 ± 0.001	0.801 ± 0.001
iSulf-Cys	10	0.656 ± 0.007	0.673 ± 0.007	0.639 ± 0.001	0.716 ± 0.009
SulCysSite	10		0.745 ± 0.006	0.744 ± 0.002	0.806 ± 0.002
Sulf_FSVM	10	0.711 ± 0.002	0.733 ± 0.004	0.708 ± 0.002	0.788 ± 0.002
LSTM_*WE*_	10	0.739 ± 0.006	0.694 ± 0.042	0.744 ± 0.008	0.800 ± 0.011
RF_*EAAC*_	10	0.733 ± 0.006	0.607 ± 0.021	0.750 ± 0.007	0.753 ± 0.006
RF_*E+S+A*_	10	0.743 ± 0.009	0.728 ± 0.027	0.745 ± 0.009	0.807 ± 0.010

*The cross-validation dataset was derived from Yang’s publication (Yang et al., 2014).

### Conservation of the CSO Modification and the Development of General LSTM_*WE*_ Models

**FIGURE 6:**
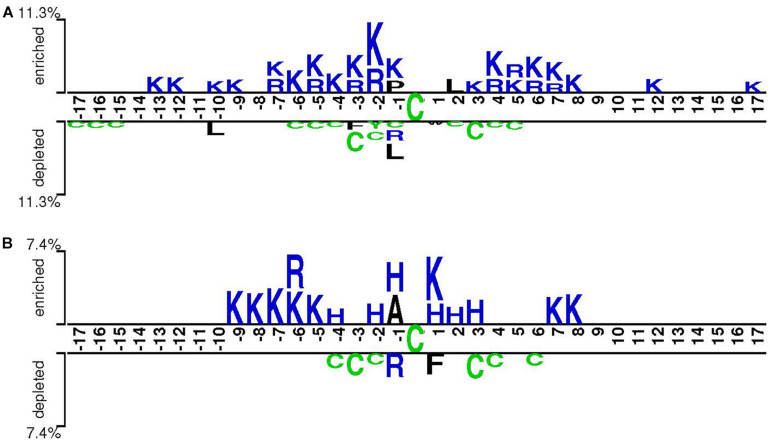
Sequence pattern surrounding the CSO sites, including the significantly enriched and depleted residues based on CSO-containing peptides and non-modification peptides for *H. sapiens* **(A)** and *A. thaliana* **(B)** (*P* < 0.05, *t*-test with Bonferroni correction). The pattern was generated using the two-sample-logo method (Vacic et al., 2006).

Cysteine S-sulphenylation has been identified across various organisms, ranging from yeasts to worms and from plants to humans (Men and Wang, 2007; Hourihan et al., 2016). To understand its conservation, we compared the characteristics of CSO-containing peptides in human and arabidopsis species, respectively, using the two-sample-logo approach (Vacic et al., 2006). Figure 6 showed that both species shared the enriched basic amino acids R and K and the depleted polar neutral amino acid C. Nevertheless, the amino acid H was enriched for *A. thaliana* whereas the hydrophobic amino acid L was depleted for *H. sapiens*. As the characteristics of CSO-containing peptides were similar between both species, we hypothesized the generalization ability of our developed models. To test this hypothesis, we used the human LSTM_*WE*_ model to predict the arabidopsis independent test dataset and employed the Arabidopsis LSTM_*WE*_ model to predict the human independent test dataset. The AUC values were 0.799 and 0.766, respectively, significantly larger than the random prediction (i.e., AUC = 0.5; Table 3). Nevertheless, the cross-species prediction had relatively low performance compared to the self-species prediction (AUC = 0.876/0.839 for arabidopsis/human, respectively). As the CSO sites were systematically analyzed in a few species, we developed a general CSO prediction model according to its conservation to boost the investigation for other species. Accordingly, we mixed the training datasets of *H. Sapiens* and *A. thaliana* and constructed the general LSTM_*WE*_ model and validated it using the independent datasets from both organisms. The performance of the general LSTM_*WE*_ model was slightly lower than that of the self-species prediction, which may be caused by the interference of the CSO characteristics of other species (Table 3). Overall, the conservation of the CSO modification leads to the effective prediction of the general LSTM_*WE*_ classifier.

**TABLE 3:** Evaluation of species-specific and general LSTM_*WE*_ models using the independent test sets from different species.

**Independent test sets**	**LSTM_*WE*_ model (AUC value)**		
	**Arabidopsis-specific**	**Human-specific**	**General**
*A. thaliana*	0.876	0.799	0.863
*H. sapiens*	0.766	0.839	0.834

**FIGURE 7:**
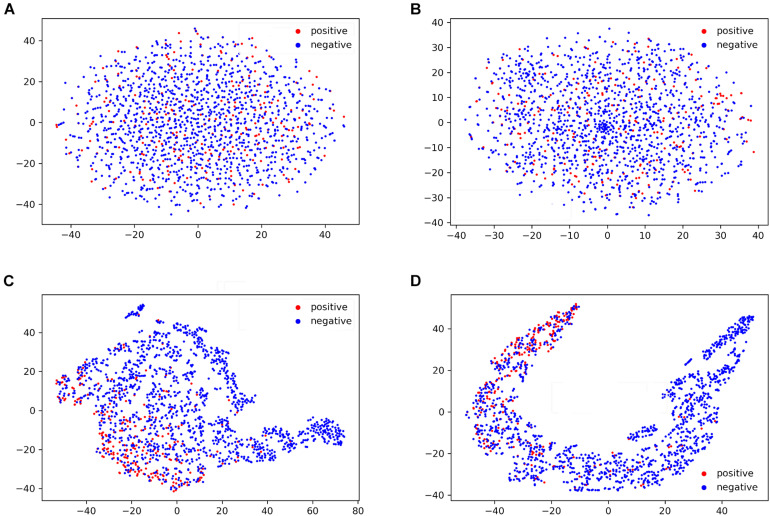
T-SNE visualization of the distributions of peptides in the human independent dataset for the outputs of input layer **(A)**, word embedding layer **(B)**, LSTM layer **(C)**, and dense layer **(D)** of the general LSTM_*WE*_ model.

To further understand the performance of the general LSTM_*WE*_ classifier, we visualized the sample distribution, based on the human independent dataset, from the outputs of the input layer, WE layer, LSTM layer, and dense layer of the general model using the t-SNE algorithm (van der Maaten and Hinton, 2008; Figure 7). After the input layer (Figure 7A), the positive and negative samples were mixed, as the training goes on (Figures 7B,C), positive and negative samples were gradually separated. After the LSTM layer, they were separated (Figure 7D). This comparison indicates that the LSTM layer is a powerful method to detect the distinctive features of the positives and negatives. A similar observation is made for the arabidopsis independent test dataset (Supplementary Figure 9).

### Construction of the Online CSO Predictor

We developed an easy-to-use online tool for the prediction of the CSO sites, dubbed DeepCSO. DeepCSO contains three LSTM_*WE*_ models: the general model and two species-specific models (i.e., *H. sapiens* and *A. thaliana*). The users could select the general model or species-specific model at the input interface and input the query protein sequences directly or upload the sequence file. After the job submission, the prediction will start and the prediction process may take several minutes. Finally, the prediction results are output in tabular form with five columns: sequence header, position, sequence, prediction score, and prediction results at the specificity levels of 80, 85, and 90%, respectively.

Several Cysteine modification types have been reported in the human organism, such as carbonylation (Wang et al., 2014; Chen et al., 2017, 2018a; Zhang S. et al., 2019), oxidation (Gupta et al., 2017; Akter et al., 2018), succination (Adam et al., 2017), and sulfenylation. Some Cysteine sites can be modified with multiple modification types, which cause PTM cross-regulation. To examine potential PTM cross-regulation at the proteome scale, we downloaded the latest human protein sequences from the Swiss-Prot database (version: 2020_05) and applied the human DeepCSO predictor to predict the potential CSO sites with the annotation of the reported Cysteine modifications (Supplementary Table 6). This resource will assist in the investigation of the Cystine co-regulation in the community.

## Conclusion

The current prediction tools for CSO sites are based on traditional ML methodology that requires experts to pre-define informative features, and no prediction tool has been developed for other than the human organism. In this study, three LSTM-based prediction models were constructed, where two were organism-specific and one was general, and they compared favorably to the reported models. Despite lacking pre-defined features, the deep learning classifier demonstrated superior performance compared to the traditional machine learning methods. This may be due to the self-learning ability of deep learning. The outstanding performance of the general model suggests that the CSO is well conserved and the LSTM-based model has an advantage in long-term memory to capture the key features of the entire sequences.

## Data Availability Statement

The 10-fold cross-validation and independent data sets can be found in http://www.bioinfogo.org/DeepCSO/.

## Author Contributions

LL conceived this project. XL and SL constructed the algorithms under the supervision of LL and YZ.; CJ, XL, and NH analyzed the data. XL, YZ, NH, and ZC, and LL wrote the manuscript. All authors read and approved the final manuscript.

## Conflict of Interest

The authors declare that the research was conducted in the absence of any commercial or financial relationships that could be construed as a potential conflict of interest.

## Footnotes

1 http://www.genome.jp/aaindex/
