# Supplementary material for: DeepCSO: A Deep-Learning Network Approach to Predicting Cysteine S-Sulphenylation Sites
Source: Front Cell Dev Biol. 2020 Dec 1;8:594587. doi: 10.3389/fcell.2020.594587 (PMC7736615; doi:10.3389/fcell.2020.594587)
Supplement: Supplementary file 2 [file Table_2.DOCX]

**Table S1.** Summary of the experientially identified CSO sites reported in the literature

| Species | Cell lines | Number of CSO sites | PMID |
| --- | --- | --- | --- |
| *Homo sapiens* | HeLa | 1098 | 30177848 |
| *Homo sapiens* | A549 | 1173 | 30177848 |
| *Homo sapiens* | RKO | 1105 | 25175731 |
| *Homo sapiens* | RKO | 1283 | 28355876 |
| *Homo sapiens* | THP-1 | 268 | 27690452 |
| *Arabidopsis thaliana* |  | 1537 | 31578252 |

**Table S2.** The AAindex properties with AUC values>0.7 using the RF classifier

| **Serial No.** | **Physiochemical property** | **AUC** |
| --- | --- | --- |
|  | HUTJ700103 | 0.750194 |
|  | HUTJ700102 | 0.746396 |
|  | FAUJ880111 | 0.744556 |
|  | RICJ880113 | 0.743962 |
|  | WILM950103 | 0.738166 |
|  | COWR900101 | 0.737601 |
|  | WILM950101 | 0.736414 |
|  | RADA880105 | 0.728701 |
|  | FINA910104 | 0.728548 |
|  | EISD860102 | 0.728289 |
|  | ZIMJ680104 | 0.728195 |
|  | ONEK900102 | 0.724703 |
|  | LEVM760101 | 0.724362 |
|  | CHOC760104 | 0.72221 |
|  | AURR980116 | 0.7202 |
|  | KLEP840101 | 0.720071 |
|  | OOBM850103 | 0.719612 |
|  | FINA910103 | 0.719424 |
|  | NAKH920108 | 0.717707 |
|  | GARJ730101 | 0.717343 |
|  | GUOD860101 | 0.715426 |
|  | FASG760103 | 0.714051 |
|  | MITS020101 | 0.712534 |
|  | FINA910102 | 0.71204 |
|  | WOLS870102 | 0.711687 |
|  | RADA880103 | 0.710135 |
|  | RADA880108 | 0.706737 |
|  | NADH010102 | 0.704762 |
|  | CHOP780207 | 0.704421 |
|  | NAKH900110 | 0.70408 |
|  | CHOP780205 | 0.703798 |
|  | DAWD720101 | 0.703751 |
|  | NADH010103 | 0.703139 |
|  | WOLS870103 | 0.700553 |
|  | LEVM760105 | 0.700494 |
|  | KIDA850101 | 0.700259 |

**Table S3. Hyper-parameters optimization scheme**

| **Grid search space** |
| --- |
| **For RF**:  param_test= {  'n_estimators':range(100,1001,20),  'max_depth':range(6,15,2),  'min_samples_split':range(2,9,1)  }  *Note: the above range was divided into three sections according to the last test results.*  gsearch= GridSearchCV(  estimator = RandomForestClassifier(oob_score=True, random_state=10),  param_grid =param_test,  n_jobs=-1,  scoring='roc_auc',  iid=False,  cv=3,  verbose=2,  refit=True  )  **For SVM**:  param_test= {  'C':range(0.5,1.0,0.25)  }  gsearch= GridSearchCV(  estimator = svm.SVC (kernel='rbf', gamma='auto', probability=True),  param_grid =param_test,  n_jobs=-1,  scoring='roc_auc',  iid=False,  cv=3,  verbose=2,  refit=True  ) |
| **Bayesian optimization space** |
| space = {  ‘window’: hp.choice(‘window’, [35,33,31,29,27,25,23]),  'emb': hp.choice('emb', [3,4,5,6,7]),  'lstm': hp.choice('lstm', [8,16,32,64]),  'filters': hp.choice('filters', [18,20,22,24]),  'kernel_size':hp.choice('kernel_size',[8,9,10,11,12]),  'units': hp.choice('units', [8,16,32,64]),  'batch_size': hp.choice('batch_size', [16,32,64,128]),  'lr_decay': hp.loguniform('lr_decay', np.log(0.000001), np.log(0.001)),  'dropout_rate': hp.uniform('dropout_rate', 0.0, 0.5),  'epochs': hp.choice('epochs', [50,60,70,80,90,100,110,120,130,140,150]),  } |

**Table S4. The optimal hyper-parameters for the DL models**

| Hyper-parameters^1^ | LSTM | | 1D-CNN | | 2D-CNN | |
| --- | --- | --- | --- | --- | --- | --- |
|  | Human | Arabidopsis | Human | Arabidopsis | Human | Arabidopsis |
| Window | 33 | 33 | 33 | 33 | 33 | 33 |
| emb | 5 | 4 | 5 | 4 | NA | NA |
| lstm^2^ | 32 | 32 | NA | NA | NA | NA |
| filters^3^ | NA | NA | 22 | 20 | 15 | 15 |
| kernel_size^3^ | NA | NA | 9 | 9 | 5×5 | 5×5 |
| pool_size^4^ | NA | NA | NA | NA | 3×3 | 3×3 |
| Units | 16 | 32 | 16 | 16 | 8 | 8 |
| batch_size | 32 | 64 | 256 | 256 | 256 | 256 |
| lr_decay | 2.98e-6 | 9.69e-6 | 1.86e-5 | 1.72e-5 | 1.82e-5 | 1.98e-5 |
| dropout_rate | 0.3 | 0.279 | 0.436 | 0.386 | 0.436 | 0.423 |
| epochs^5^ | 50 | 50 | 60 | 50 | 60 | 55 |

^1^ Window: sequence length; emb: the dimension of the dense embedding; lstm: the dimension of the output space of LSTM layer; filters: the dimension of the output space (i.e. the number of output filters in the convolution); kernel_size: the length of the convolution window; pool_size: the length of the pooling window; units: the dimension of the output space of dense layer; batch_size: number of samples per gradient update; lr_decay: The exponential decay rate for the first moment estimates; dropout_rate: fraction (ranging from 0 to 1) of the units to drop for the linear transformation of the inputs; epochs: Number of epochs to train the model; NA: not available.

^2^the parameter lstm is specific to the LSTM models.

^3^ the parameters filters and kernel_size are specific to the CNN models.

^4^ the parameters pool_size is specific to the 2D-CNN models.

^5^ the parameter epoch stores the maximum of epochs designed for early-stop strategy.

**Table S5. Performances of various classifiers for *A. thaliana* and *H. sapiens* in terms of the independent test**

| ***Arabidopsis thaliana*** | | | | | | |
| --- | --- | --- | --- | --- | --- | --- |
| **Classifier^1^** | **ACC^2^** | **Sn^2^** | **Sp^2^** | **MCC2^2^** | **AUC^2^** | **AUC01^2^** |
| RF_BINARY_ | 0.753±0.005 | 0.498±0.031 | 0.800±0.000 | 0.251±0.024 | 0.739±0.010 | 0.014±0.002 |
| RF_EAAC_ | 0.780±0.004 | 0.672±0.022 | 0.800±0.000 | 0.385±0.017 | 0.819±0.004 | 0.027±0.002 |
| RF_WE_ | 0.752±0.007 | 0.493±0.043 | 0.800±0.000 | 0.247±0.034 | 0.705±0.157 | 0.017±0.002 |
| RF_AAINDEX_ | 0.753±0.006 | 0.500±0.038 | 0.800±0.000 | 0.253±0.030 | 0.752±0.007 | 0.017±0.002 |
| RF_CKSAAP_ | 0.750±0.004 | 0.483±0.026 | 0.800±0.000 | 0.239±0.020 | 0.742±0.007 | 0.015±0.002 |
| RF_PSSM_ | 0.735±0.005 | 0.390±0.031 | 0.800±0.000 | 0.164±0.026 | 0.690±0.006 | 0.011±0.001 |
| RF_E+S+A_ | 0.772±0.005 | 0.622±0.030 | 0.800±0.000 | 0.348±0.023 | 0.811±0.007 | 0.024±0.002 |
| SVM_BINARY_ | 0.758±0.004 | 0.535±0.025 | 0.800±0.000 | 0.281±0.020 | 0.735±0.007 | 0.020±0.002 |
| SVM_EAAC_ | 0.753±0.006 | 0.502±0.037 | 0.800±0.000 | 0.254±0.029 | 0.723±0.012 | 0.017±0.002 |
| SVM_AAINDEX_ | 0.762±0.005 | 0.559±0.030 | 0.800±0.000 | 0.299±0.023 | 0.767±0.012 | 0.022±0.002 |
| SVM_CKSAAP_ | 0.749±0.005 | 0.477±0,030 | 0.800±0.000 | 0.235±0.024 | 0.695±0.024 | 0.015±0.003 |
| SVM_PSSM_ | 0.729±0.003 | 0.352±0.020 | 0.800±0.000 | 0.132±0.017 | 0.596±0.015 | 0.012±0.001 |
| 2D-CNN_PSSM_ | 0.764±0.002 | 0.570±0.016 | 0.800±0.000 | 0.308±0.012 | 0.765±0.006 | 0.022±0.002 |
| 1D-CNN_WE_ | 0.791±0.005 | 0.741±0.034 | 0.800±0.000 | 0.436±0.024 | 0.850±0.011 | 0.028±0.003 |
| **LSTM_WE_** | **0.796±0.005** | **0.776±0.032** | **0.800±0.000** | **0.462±0.024** | **0.863±0.008** | **0.032±0.003** |
| ***Homo sapiens*** | | | | | | |
| **Classifier^1^** | **ACC^2^** | **Sn^2^** | **Sp^2^** | **MCC^2^** | **AUC^2^** | **AUC01^2^** |
| RF_BINARY_ | 0.750±0.002 | 0.477±0.015 | 0.800±0.001 | 0.233±0.012 | 0.729±0.003 | 0.016±0.001 |
| RF_EAAC_ | 0.773±0.003 | 0.626±0.016 | 0.800±0.000 | 0.348±0.012 | 0.807±0.001 | 0.024±0.001 |
| RF_WE_ | 0.746±0.003 | 0.454±0.018 | 0.800±0.000 | 0.215±0.014 | 0.724±0.002 | 0.016±0.001 |
| RF_AAINDEX_ | 0.749±0.002 | 0.471±0.010 | 0.800±0.000 | 0.228±0.008 | 0.728±0.003 | 0.016±0.001 |
| RF_CKSAAP_ | 0.762±0.004 | 0.553±0.024 | 0.800±0.000 | 0.293±0.019 | 0.764±0.003 | 0.017±0.001 |
| RF_PSSM_ | 0.746±0.003 | 0.448±0.019 | 0.800±0.000 | 0.210±0.016 | 0.699±0.003 | 0.014±0.001 |
| RF_E+S+A_ | 0.766±0.003 | 0.580±0.020 | 0.800±0.000 | 0.314±0.015 | 0.782±0.004 | 0.021±0.001 |
| SVM_BINARY_ | 0.745±0.003 | 0.448±0.021 | 0.800±0.000 | 0.210±0.017 | 0.709±0.004 | 0.015±0.001 |
| SVM_EAAC_ | 0.736±0.006 | 0.386±0.037 | 0.800±0.000 | 0.160±0.030 | 0.667±0.013 | 0.012±0.002 |
| SVM_AAINDEX_ | 0.747±0.002 | 0.460±0.016 | 0.800±0.000 | 0.220±0.013 | 0.716±0.006 | 0.016±0.001 |
| SVM_CKSAAP_ | 0.737±0.005 | 0.393±0.033 | 0.800±0.000 | 0.165±0.027 | 0.670±0.017 | 0.011±0.002 |
| SVM_PSSM_ | 0.724±0.003 | 0.306±0.019 | 0.800±0.000 | 0.093±0.016 | 0.553±0.016 | 0.011±0.001 |
| 2D-CNN_PSSM_ | 0.764±0.002 | 0.566±0.015 | 0.800±0.000 | 0.303±0.012 | 0.760±0.003 | 0.018±0.001 |
| 1D-CNN_WE_ | 0.783±0.002 | 0.693±0.012 | 0.800±0.000 | 0.398±0.009 | 0.830±0.005 | 0.026±0.002 |
| **LSTM_WE_** | **0.783±0.003** | **0.693±0.021** | **0.800±0.000** | **0.399±0.015** | **0.831±0.003** | **0.027±0.001** |

*Note*: ^1^ The RF classifiers with the different features were named as RF_BINARY_, RF_WE_, etc. The 1D CNN and LSTM classifiers with the word embedding approach were named as 1D-CNN_WE_ and LSTM_WE_, respectively.^2^ ACC, Sn, Sp, MCC, AUC and AUC01 were described in Materials and Methods. In the ten-fold cross-validation, ten models were constructed using the ten different validation datasets. The average performance and standard deviation of the ten models were calculated for the independent dataset.


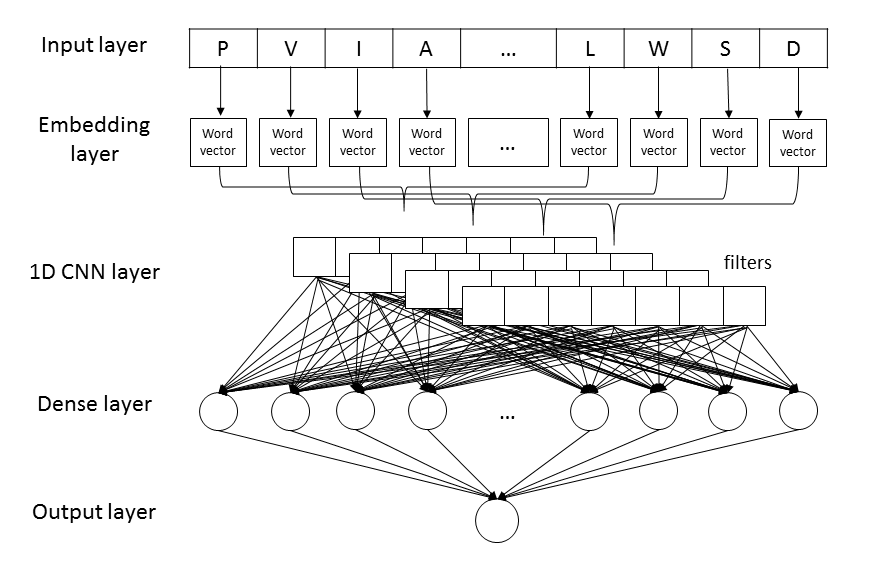


**Figure S1.** The 1D-CNN_WE_ architecture.


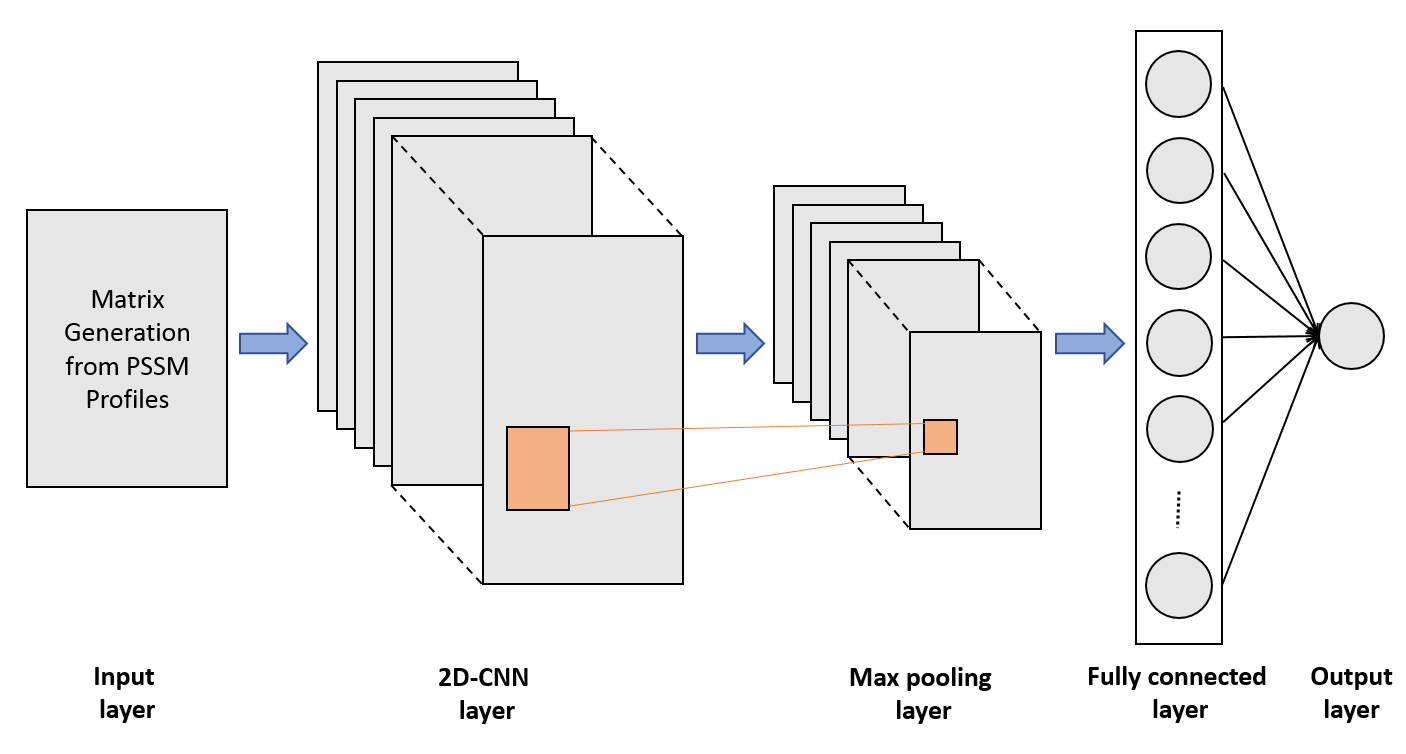


**Figure S2.** The 2D-CNN_PSSM_ architecture.


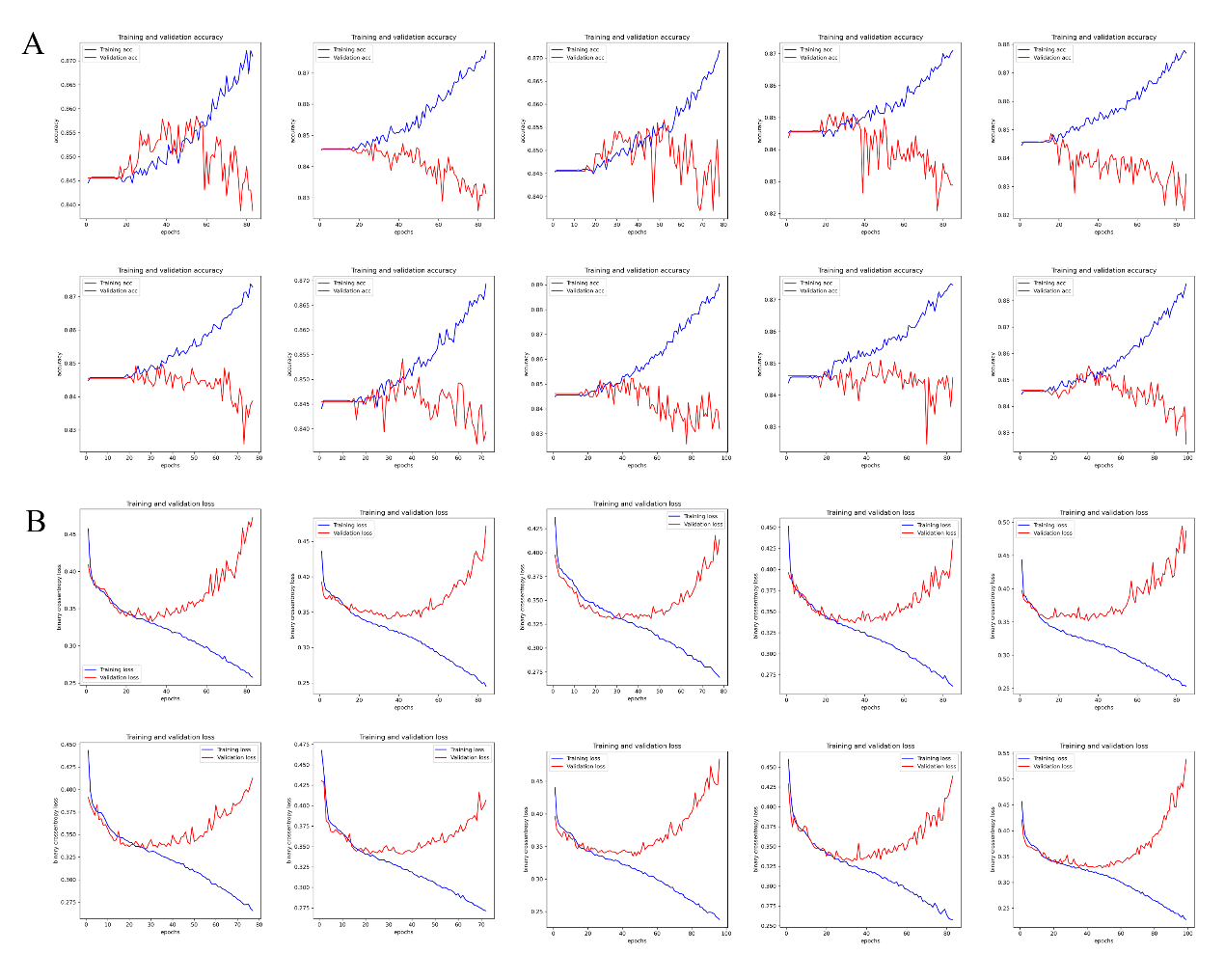


**Figure S3.** The training and validation accuracy curves (A) and loss curves (B) of the humans LSTM_WE_ model for ten-fold cross-validation. The training curves were coloured blue and the validation curves were coloured red.


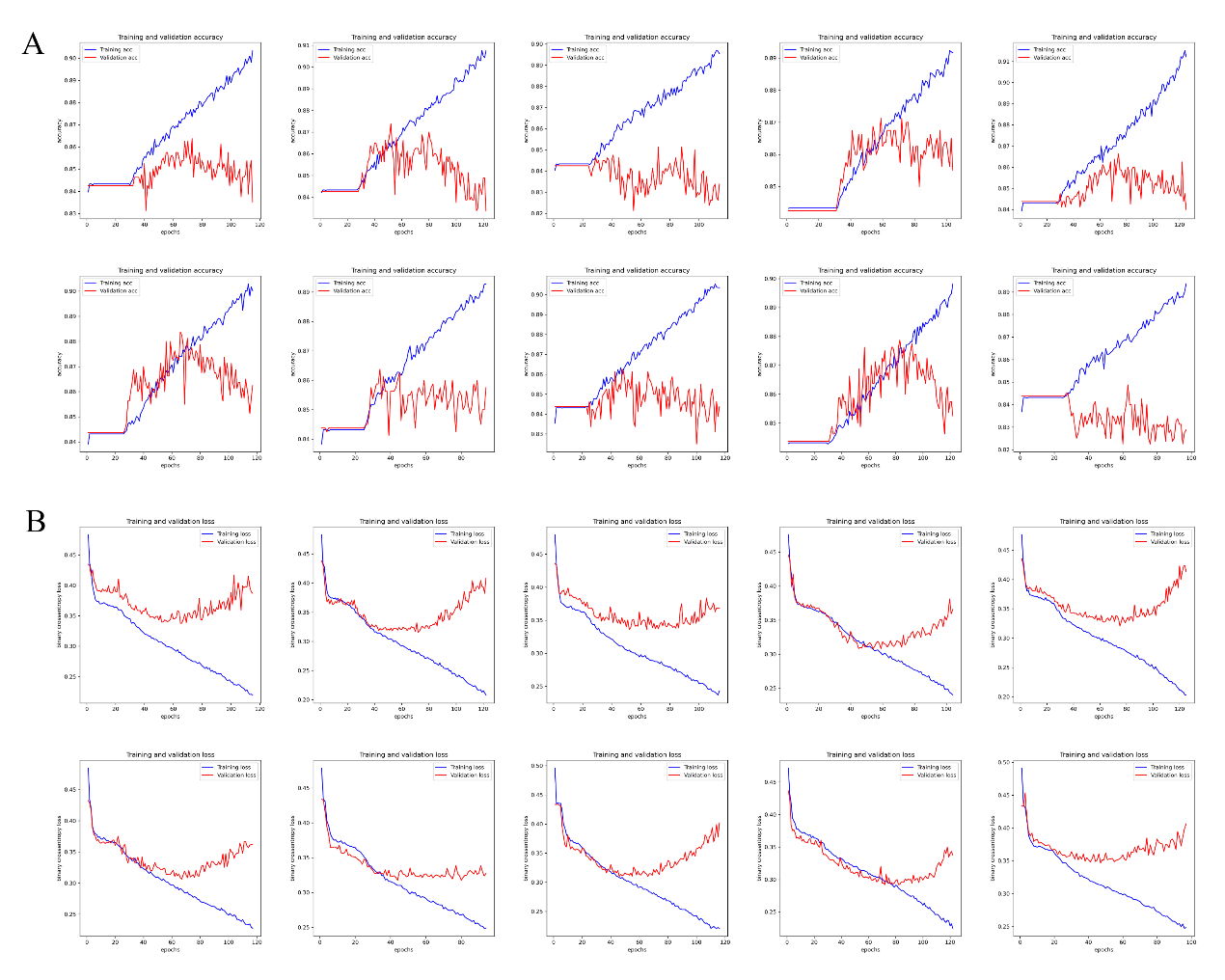


**Figure S4.** The training and validation accuracy curves (A) and loss curves (B) of the Arabidopsis LSTM_WE_ model for ten-fold cross-validation. The training curves were colored blue and the validation curves were colored red.


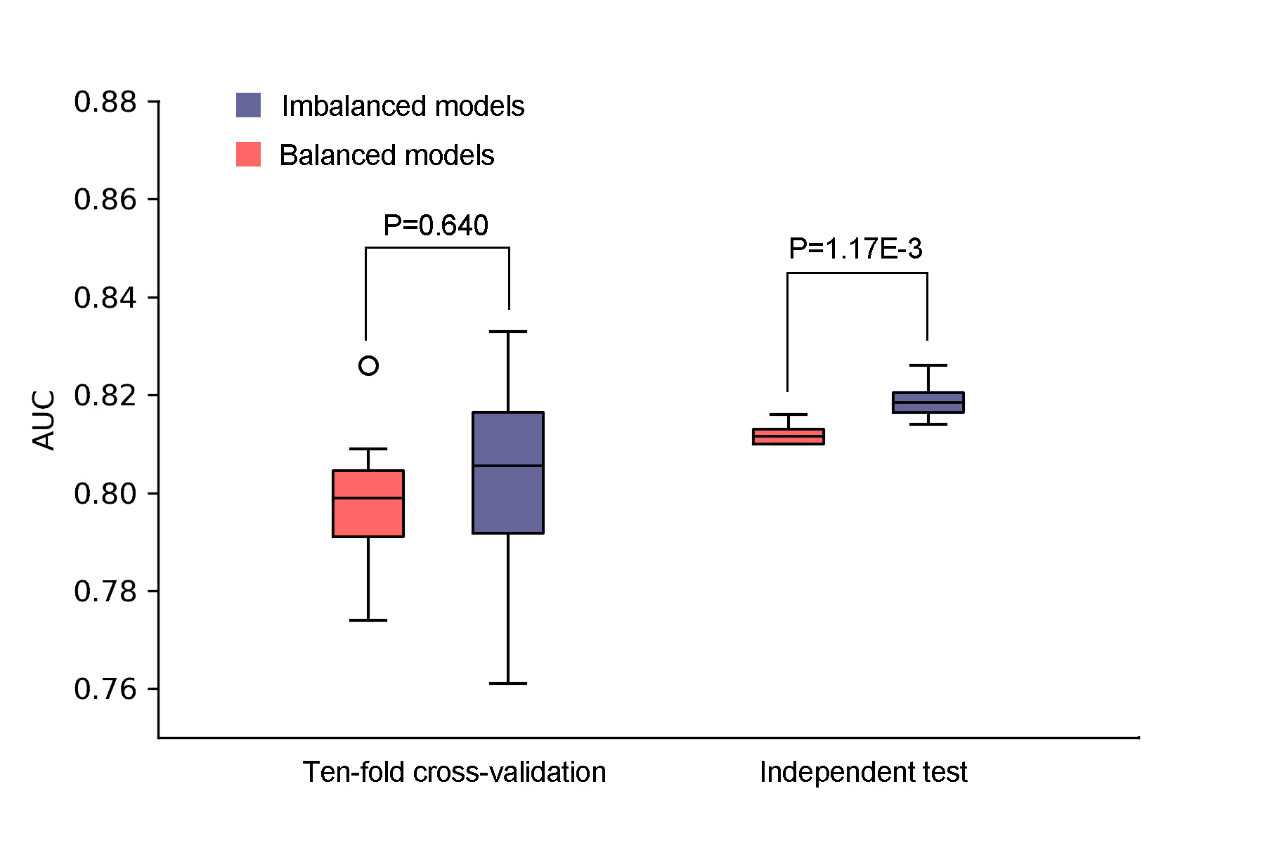


**Figure S5.** Performance comparisons between the RF_EAAC_ models constructed using a balanced dataset and an imbalanced dataset from the *Arabidopsis* data in terms of ten-fold cross-validation and the independent test.


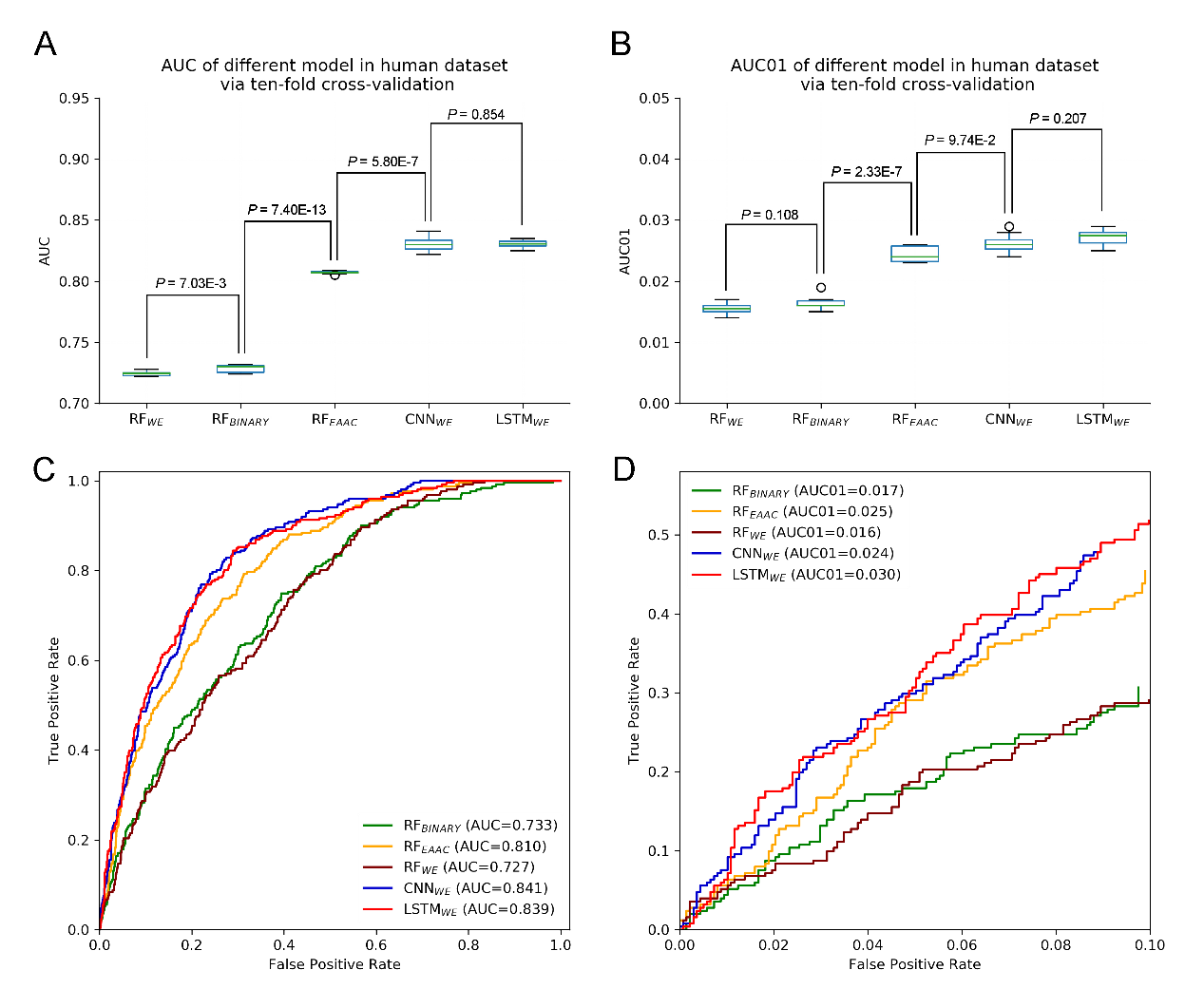


**Figure S6.** Performance comparison of different CSO predictors on *Homo sapiens*. The performances of CSO predictors were compared in terms of AUC (A) and AUC01 (B), respectively, for ten-fold cross-validation. AUC (C) and AUC01 (D) curves were generated using the independent test.


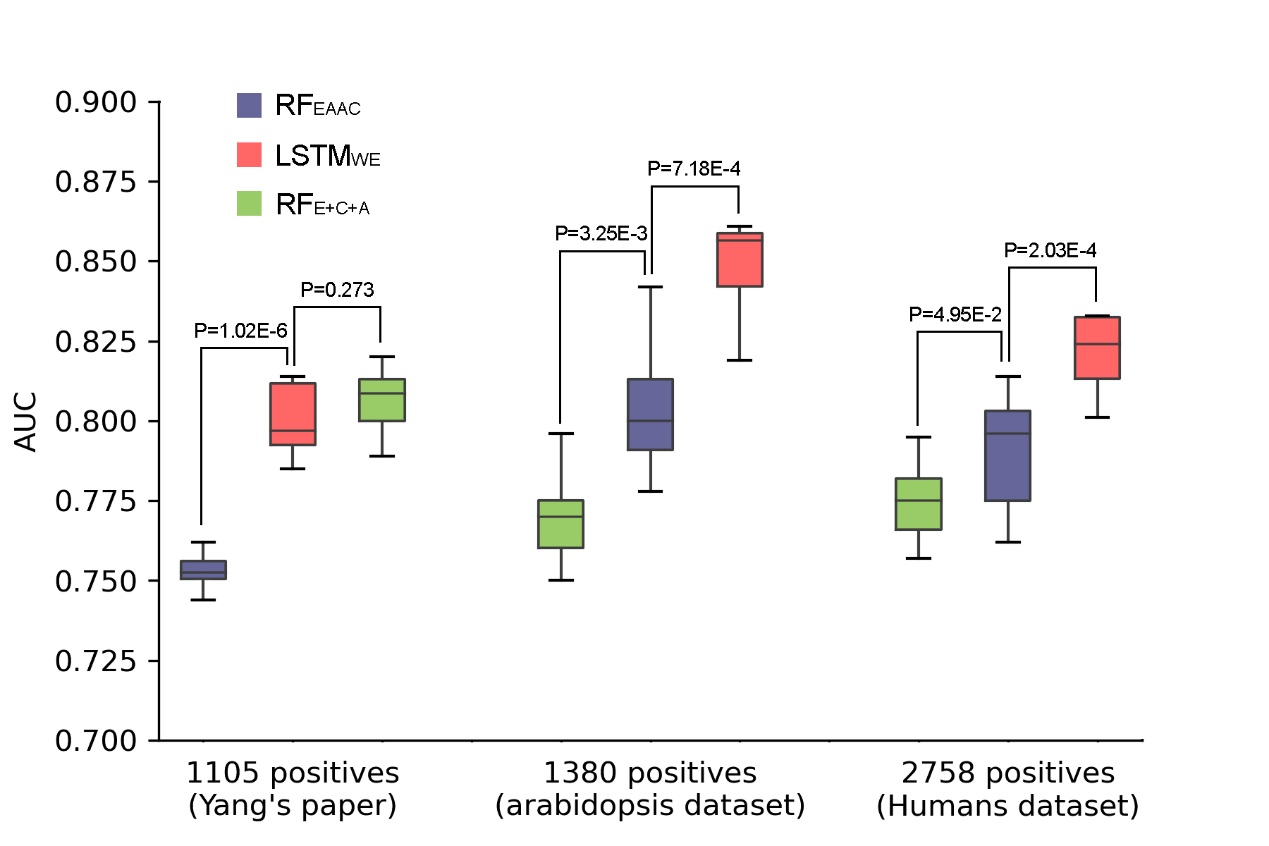


**Figure S7.** Performance comparison among different models for different sizes of training data.

**
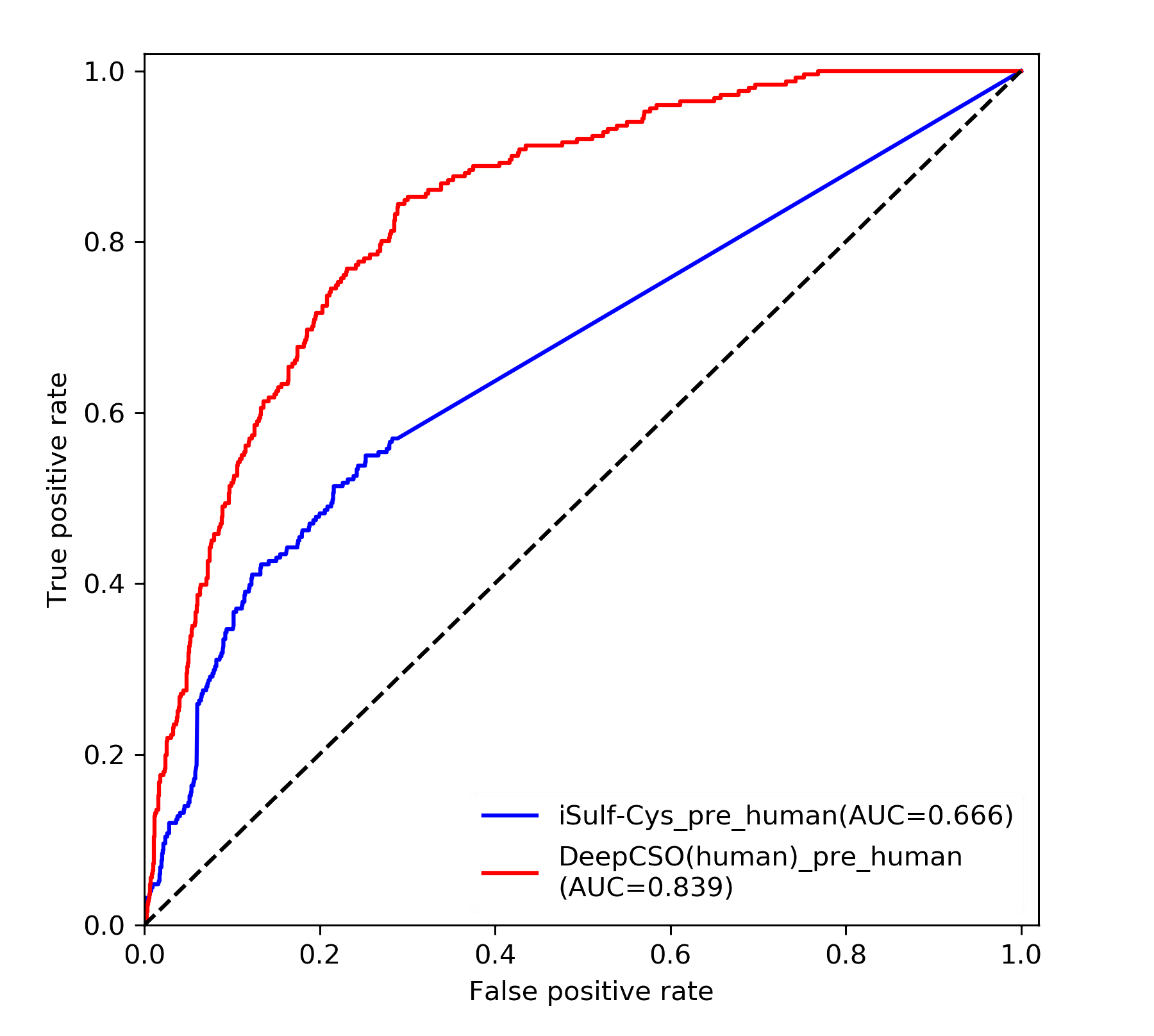
Figure S8.** Performance comparison between LSTM_WE_ and iSulf-Cys in terms of the human independent dataset.


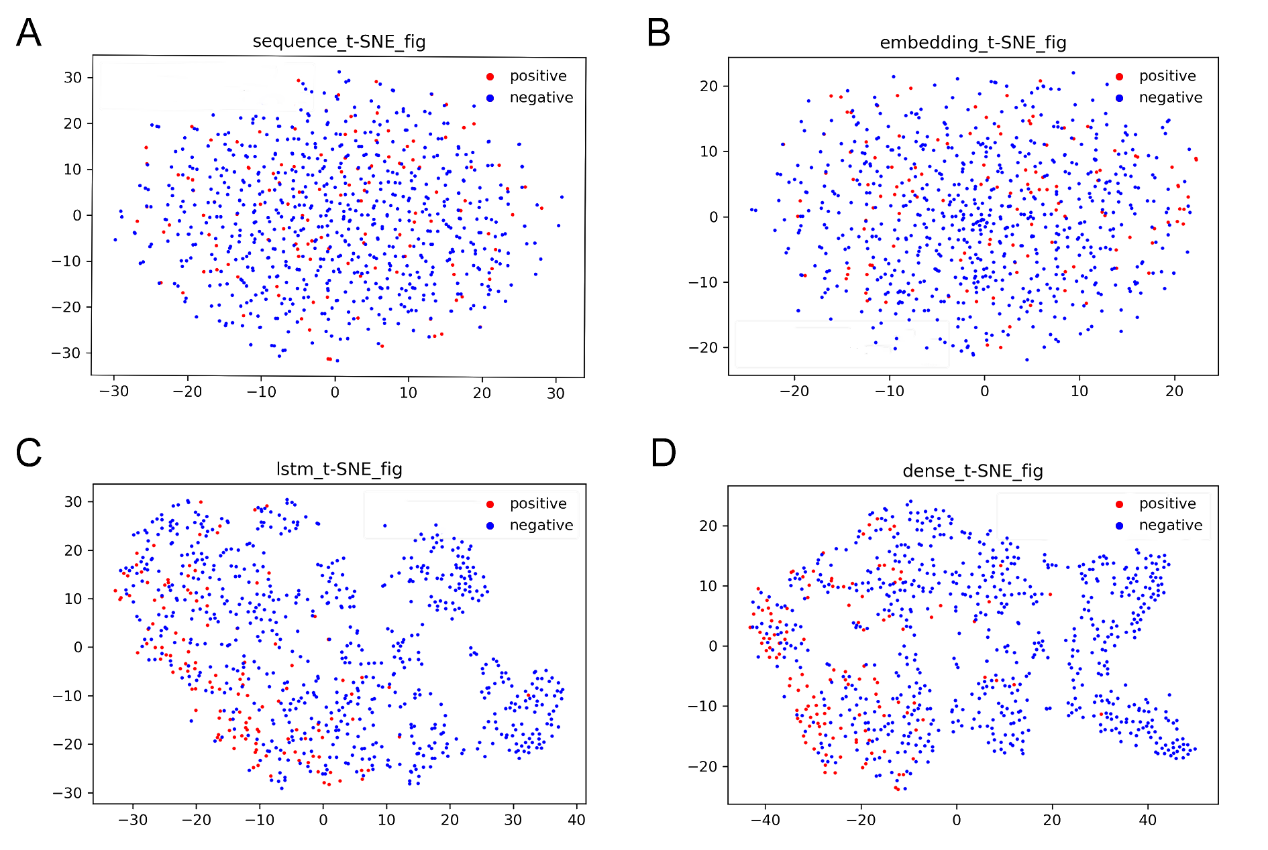


**Figure S9.** T-SNE visualization of the distributions of peptides in the *A. thaliana* independent dataset for the outputs of the input layer (A), embedding layer (B), LSTM layer (C) and dense layer (D) of the general LSTM_WE_ model.
